# Monolayer-Based Single-Photon Source in a Liquid-Helium-Free Open Cavity Featuring 65% Brightness and Quantum Coherence

**DOI:** 10.1021/acs.nanolett.3c02584

**Published:** 2023-09-09

**Authors:** Jens-Christian Drawer, Victor Nikolaevich Mitryakhin, Hangyong Shan, Sven Stephan, Moritz Gittinger, Lukas Lackner, Bo Han, Gilbert Leibeling, Falk Eilenberger, Rounak Banerjee, Sefaattin Tongay, Kenji Watanabe, Takashi Taniguchi, Christoph Lienau, Martin Silies, Carlos Anton-Solanas, Martin Esmann, Christian Schneider

**Affiliations:** † Institute of Physics, Carl von Ossietzky University Oldenburg, 26129 Oldenburg, Germany; ‡ University of Applied Sciences Emden/Leer, 26723 Emden, Germany; § Institute of Applied Physics, Abbe Center of Photonics, Friedrich Schiller University Jena, 07743 Jena, Germany; ∥ Fraunhofer-Institute for Applied Optics and Precision Engineering IOF, 07743 Jena, Germany; ⊥ Max-Planck-School of Photonics, 07743 Jena, Germany; # Materials Science and Engineering, School for Engineering of Matter, Transport, and Energy, Arizona State University, Tempe, Arizona 85287, United States; ∇ Research Center for Functional Materials, National Institute for Materials Science, 1-1 Namiki, Tsukuba 305-0044, Japan; ○ International Center for Materials Nanoarchitectonics, National Institute for Materials Science, 1-1 Namiki, Tsukuba 305-0044, Japan; ● Depto. de Física de Materiales, Instituto Nicolás Cabrera, Instituto de Física de la Materia Condensada, Universidad Autónoma de Madrid, 28049 Madrid, Spain

**Keywords:** two-dimensional materials, quantum dots, single-photon source, open microcavity

## Abstract

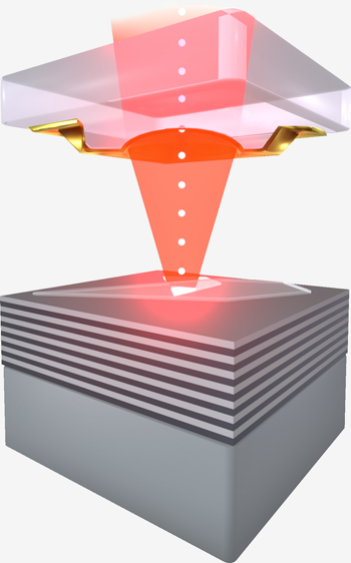

Solid-state single-photon sources are central building blocks in quantum information processing. Atomically thin crystals have emerged as sources of nonclassical light; however, they perform below the state-of-the-art devices based on volume crystals. Here, we implement a bright single-photon source based on an atomically thin sheet of WSe_2_ coupled to a tunable optical cavity in a liquid-helium-free cryostat without the further need for active stabilization. Its performance is characterized by high single-photon purity (g^(2)^(0) = 4.7 ± 0.7%) and record-high, first-lens brightness of linearly polarized photons of 65 ± 4%, representing a decisive step toward real-world quantum applications. The high performance of our devices allows us to observe two-photon interference in a Hong–Ou–Mandel experiment with 2% visibility limited by the emitter coherence time and setup resolution. Our results thus demonstrate that the combination of the unique properties of two-dimensional materials and versatile open cavities emerges as an inspiring avenue for novel quantum optoelectronic devices.

Solid-state single-photon sources are devices of central importance to enable scalable quantum optical applications. They play a pivotal role in quantum communication, metrology, and quantum computing.^1−5^ As such, it is crucial to engineer and characterize these devices according to their requirements in these real-life applications. For the vast majority of such applications, three performance parameters of a single-photon source are of exceptional importance: the single-photon purity, which is characterized via the second-order autocorrelation function *g*^(2)^(0), the first lens brightness, which reflects the probability of a single photon emission from the device following an excitation, and finally the capability of the emitted single photons to display quantum interference.^3^ In addition, the scalable and cost-effective implementation of such devices with top performance is highly desirable but thus far elusive.

In recent years, a large palette of solid-state single-photon emitters has emerged, featuring different degrees of material-processing versatility and a wide range of emission wavelengths, operation temperatures, and polarization properties.^6^ Single InAs quantum dots, which are coupled to photonic cavities, have advanced as the most mature solid-state platform; however, their cost-effective implementation is strongly hindered by expensive growth and nanofabrication routines, and their recent implementation in deterministic tunable cavities still relies on nonsustainable liquid-helium cryostats.

Atomically thin semiconductors, on the other hand, are promising candidates for optoelectronic and quantum applications:^7,8^ They combine a low-cost synthesis with maximum compatibility in terms of integration into heterostructures. An example of this class of ultimately thin materials is the inorganic transition-metal dichalcogenide (TMDC) WSe_2_, which features single-photon emission from tightly localized excitons in monolayers at cryogenic temperatures.^9−13^ TMDC single-photon emitters provide high quantum efficiency,^14−16^ charge tunability,^11^ and polarization control,^17,18^ and most notably, they can be seeded at precise locations by engineering local mechanical strain in the monolayer.^19,20^

The success of solid-state single-photon emitters in general relies on photonic cavities to shape the optical density of states around the emitter, i.e. increasing the spontaneous emission rate via the Purcell effect into a specific photonic mode, ensuring optimal light collection.^21^ The scalable and deterministic integration of solid-state quantum emitters into photonic microcavities in general and TMDC QDs in particular is still one of the most delicate tasks in quantum engineering. While techniques based on combining nanolithography, nanoimaging, and emitter site control have been widely explored to integrate III/V QDs^22−24^ and TMDC QDs into optical resonators, more powerful and versatile approaches were recently developed.

Among those, the concept of open photonic cavities represents an *ad hoc*, fully deterministic approach for interfacing a microcavity with single-photon emitters in two-dimensional materials. In these reconfigurable Fabry–Perot resonators, the two opposing mirrors allow relative displacements in three dimensions of space, facilitating precise control of quantum light emission.^25−29^ Open-cavity-based solid-state single photon sources have so far been implemented in liquid-helium bath cryostats or in highly engineered cavity platforms requiring active phase-locking of the cavity length and fiber-based mirrors.^30−36^

In this work, we demonstrate a high-performance single-photon source based on a WSe_2_ monolayer QD that is deterministically coupled to the optical resonance of an open cavity. The reconfigurable open cavity is implemented in a low-vibration, helium-free cryostat without any active stabilization. Additionally, the cavity modes are inscribed in planar mirrors via focused ion beam (FIB) milling, providing superior control on the photonic mode engineering, as opposed to fiber-based cavities. The photonic resonator tunability allows us to deterministically position the single emitter of a wrinkled monolayer at the cavity center and tune the cavity resonance to the corresponding photon emission wavelength. Our single-photon source displays a high single-photon purity with a *g*^(2)^(0) value as low as 4.7 ± 0.7% and a first-lens brightness as high as 65%, which translates to a single-photon emission rate of 49.8 MHz utilizing a pump laser of 76.2 MHz. It furthermore displays statistically significant signs of quantum coherence in Hong–Ou–Mandel experiments with 2% interference visibility limited by the emitter dephasing time of 23.1 ps and the temporal resolution of our measurement apparatus.

The design of the open cavity sample is graphically sketched in Figure 1a. It is based on an asymmetrical mirror design to enhance the single-photon collection in the same direction as the excitation: the bottom part of the cavity consists of a distributed Bragg reflector (DBR) with a high reflectivity hosting the monolayer flake. The monolayer is capped by a thin layer of hexagonal boron nitride, guaranteeing spectral stability. The top part of the cavity is built from a glass mesa containing concave hemispherical indentations of different diameters; a 33 nm thick layer of gold is evaporated onto this structure to finalize the top mirror (for further details on sample preparation see Section S1 in the Supporting Information). A scanning electron microscope (SEM) image of the cavity top mirror (before the gold coating) is shown in Figure 1b (top). The hexagonal boron nitride capped WSe_2_ monolayer flake on the DBR is shown in Figure 1b (bottom). We placed the open-cavity device inside a low-vibration closed-cycle exchange-gas cryostat and kept it at 3.2 K. With the recent dramatic increase in helium prices, sustainable solutions not relying on liquid helium bath cryostats have become an urgent need for the quantum photonics community. However, especially the performance of spectrally tunable open cavities thus far has relied on their implementation in a vibration-free bath cryostat. Our implementation geometry of the cavity, which is sufficiently robust to not be impeded in its performance by the pulse-tube cooler of our exchange-gas cryostat, can be found in Figure S1 of the Supporting Information.

**Figure 1:**
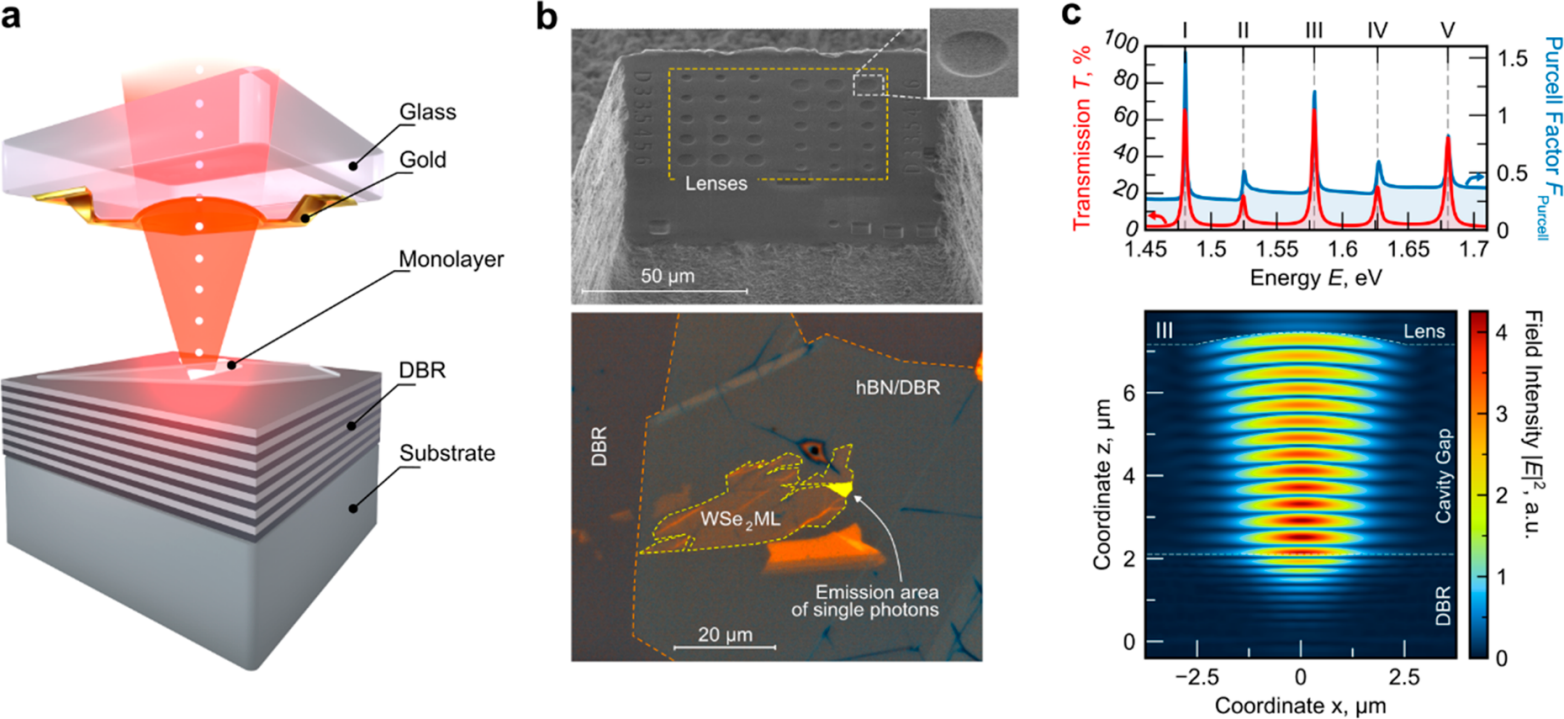
WSe_2_ monolayer in an open cavity. (a) Graphical representation of single-photon emission from a monolayer source in a plano-convex open cavity under optical excitation. The relative position of the top and bottom mirrors is adjustable by nanopositioners. (b) (top) Scanning electron microscope image of the mesa-type cavity top mirror with hemispherical indentations of different diameters etched by focused ion beam lithography (before gold layer deposition) and (bottom) optical microscope image of the WSe_2_ monolayer placed on a SiO_2_/TiO_2_ DBR. The single-photon source is located at the edge of the flake near a wrinkle. (c) (top) Transmission and Purcell factor (red and blue lines) of the open-cavity system used in the experiments derived using FDTD simulation of the electric field of a dipole located at the monolayer position and (bottom) real space intensity distribution inside the cavity. The surface of the top and bottom mirror is indicated by dashed white lines.

To assess the possible performance of our cavity device, we performed finite-difference time-domain (FDTD) simulations of the experimental resonator configuration. Figure 1c (bottom) shows the resonant real space intensity distribution inside the open cavity at a wavelength of 786 nm. For a lens diameter of 5 μm and a depth of 300 nm (corresponding to a radius of curvature of 6.8 μm), the field is laterally confined to a diameter of ∼2 μm at the emitter position. Figure 1c (top) shows the calculated transmission through the top mirror for a point dipole source and the corresponding Purcell factor as a function of wavelength.

Interestingly, the simulation predicts an on-resonant Purcell enhancement of up to 1.5 (blue line in Figure 1c), in conjunction with an off-resonant suppression of spontaneous emission up to a factor of 3.8. The latter is a clear indicator of the strong suppression of so-called leaky modes in our cavity implementation. From our simulation, we can directly anticipate photon extraction efficiencies (also referred to as “first-lens brightness”) beyond 65%, under the precondition that the internal quantum efficiency of the emitter approaches unity.

The experimentally studied quantum dot (QD) like emitter, which evolves in our WSe_2_ monolayer, emerges at an emission energy of 1.5707 eV (789.3 nm). It is interesting to note that this wavelength, which is widely tunable via piezo strain,^37^ is very close to the technologically relevant Rb-87-D2 line, with the potential for a quantum memory in future repeater networks.^38^ Moreover, the emission wavelength is also compatible with free-space quantum communication applications.^39^ The spectral line width of the QD is limited by the resolution of our detection system of 200 μeV (see Figure S5 of the Supporting Information for a high-resolution spectrum). As a first important parameter of our source, a polarization-resolved measurement, carried out without the top mirror of the cavity (Figure 2a), reveals that our QD emitter displays close to perfect linear polarization up to a degree of 96.8 ± 2.5%. We attribute this remarkable feature to the emergence of the studied QD from a monolayer wrinkle,^17^ creating a local and quasi-one-dimensional strain potential^40,41^ which results in a strongly aligned dipole. As a next step, we added the top mirror and studied the performance of the coupled cavity–emitter system. We first use nonresonant continuous wave laser excitation (532 nm) and record the sample PL for a continuously varying cavity length. The resulting color heat map is plotted in Figure 2b. In our experiment, we observe longitudinal mode families, each consisting of three transverse modes, separated by a spacing of 26.3 meV. The modes are visualized by the guide to the eye in Figure 2b and emphasized in a logarithmic representation in Section S3 of the Supporting Information.

**Figure 2:**
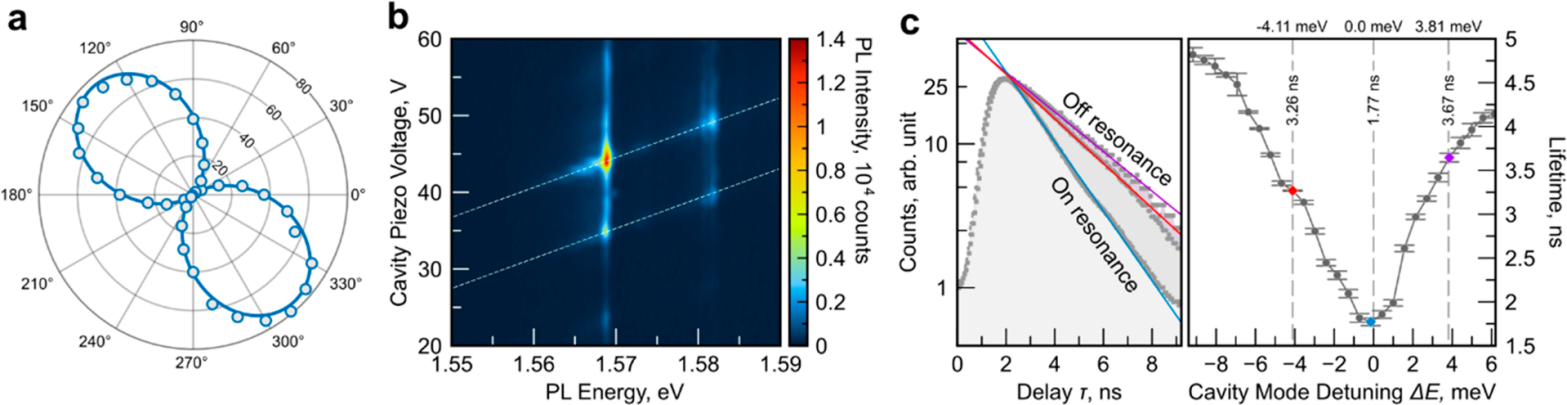
Cavity mode detuning dependent PL and lifetime. (a) Polar plot of polarization-resolved PL intensity of the emission under 532 nm continuous wave excitation. The sinusoidal fit reveals a degree of linear polarization of 96.8 ± 2.5%. (b) Color map of PL spectra when tuning the cavity optical length while the sample is strongly excited above the band gap and outside of the stopband of the microcavity by a 532 nm continuous wave laser. Cavity modes are highlighted by dashed lines. (c) (left) Lifetime in on- and off-resonant cases (blue, on resonance; red, −4.11 meV detuning; purple, +3.81 meV detuning) and (right) cavity mode detuning dependent radiative lifetime. Error bars in the right panel represent the standard error resulting from fitting the time-resolved photoluminescence data (as shown in the panel on the left) with an exponential decay function. The fitting method utilizes a damped least-squares algorithm.

The quality factor of these cavity resonances is around 600 for the chosen mirror separations of ∼5.5 μm (the separation is directly extracted from the longitudinal mode spacing). Importantly, and as reflected in the simulation in Figure 1c, the mode of lowest transverse order is a Gaussian mode, which is optimally suited for coupling to a commercial single-mode fiber.

The open nature of the cavity allows us, in a straightforward manner, to study the coupled cavity–emitter system under various detunings by changing the resonance condition via the cavity resonator length. As reflected in Figure 2b, on resonance, the photoluminescence intensity of the emitter is enhanced by more than a factor of 10, which clearly reflects the strong impact of the resonator structure on the performance of the coupled emitter–cavity system. To further improve the performance of our source, we optimize the photon injection efficiency into our WSe_2_ QD by choosing a double-resonance condition (see Figure S4 in the Supporting Information). While we maintain the spectral resonance condition between the emitter zero-phonon line and the cavity mode, we tune our pulsed excitation laser (2 ps pulse length, 76.2 MHz repetition rate) on resonance to the next higher order longitudinal cavity mode spectrally located at 740 nm with identical (lowest) transverse order. This condition allows us to inject light efficiently into the cavity to pump the emitter quasi-resonantly into a higher resonance shell.^13^ It is also important to note that quasi-resonant pumping of WSe_2_ emitters below the free exciton resonance is very important to guarantee a high quantum efficiency, which can otherwise be impeded via nonradiative losses into the free exciton bath.

To quantify the enhancement of spontaneous emission in our device more rigorously, we performed time-resolved photoluminescence measurements under varying emitter–cavity detunings. For these experiments, the QD emission line has been optically filtered via a coarse bandpass (∼2.5 meV bandwidth) and was directly detected by an avalanche photodiode connected to a time correlator. The corresponding decay dynamics for the off- and on-resonant cases are shown in Figure 2c (left). The characteristic decay times have been fitted with a single-exponential decay function and unambiguously reflect the speeding up of the spontaneous emission rate in the resonant case. The overall detuning dependence of the spontaneous emission decay is plotted in Figure 2c (right), reflecting the interplay of the spontaneous emission rate with the optical resonance bandwidth. In addition, we have also analyzed the lifetime of our emitter without the top mirror, yielding a decay time of 2.3 ns. The values in Figure 2c, therefore, indicate a cavity-induced reduction in lifetime of 25% on resonance, whereas off resonance the emitter experiences a more than 2-fold inhibition of spontaneous emission due to the presence of the open cavity. This observation is in excellent agreement with the theoretically predicted changes in the lifetime shown in Figure 1c. The predicted ratio of enhanced to inhibited emission is 3, whereas in the experiment we find 2.71 ± 0.08.

To assess the purity of the single-photon pulses emitted by our device, we measured the second-order correlation function via a standard Hanbury–Brown–Twiss (HBT) setting. From the correlation histogram in Figure 3a, we can extract a photon antibunching of *g*^(2)^ = 4.7 ± 0.7% (details on the analysis can be found in the Supporting Information). It is worth noting that in these experiments the emission was only filtered by a coarse bandpass (∼2.5 meV bandwidth); this further emphasizes the emission purity of the open-cavity device.

**Figure 3:**
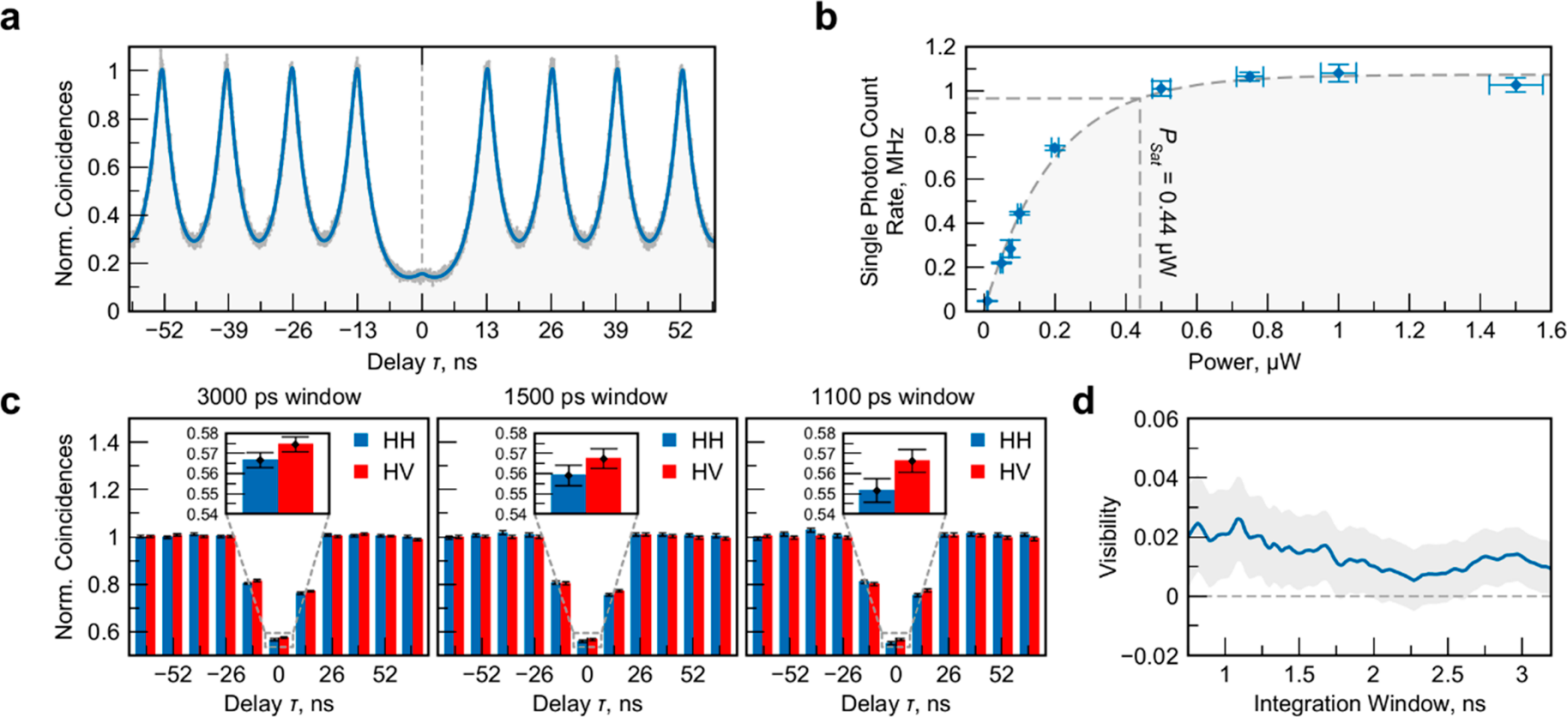
Single-photon source characterization. (a) Second-order autocorrelation function of single photons measured in an HBT experiment with 76.2 MHz pulsed excitation in the saturation regime. The data are fitted by a double-exponential decay convoluted with the system response function (details in the Supporting Information). (b) Brightness of the source as a function of optical pump power measured before focusing on the sample. Errors are the standard errors as the result of averaging over 10 samples for each point. (c) Second-order correlation function of single photons in an HOM setup discretized by the pulsed excitation for three different temporal postselection window sizes in the case of parallel (HH) and perpendicular (HV) polarization. Error bars show standard deviations of the assumed underlying Poissonian distributions of counts in each integrated window. (d) HOM interference visibility for varying temporal postselection window size. The shaded area shows the error bounds.

A critical parameter in the performance of single-photon sources is the probability of delivering a single-photon state per excitation pulse, which is usually benchmarked by the brightness at the first collection lens. To quantify this critical performance indicator, which is of central importance for quantum communication implementations, we studied the emission flux of the single-photon sources as a function of the pulsed pump power (76.2 MHz repetition rate). As shown in Figure 3b, we detect more than 1 MHz of single-photon counts in our single-photon detectors. After carefully assessing the transmission and detection efficiencies of our collection setup (see Table S1 of the Supporting Information), this value directly translates into an emission frequency of 49.8 MHz single photons that are emitted from our source and into a record first-lens brightness of 65 ± 4% in a linearly polarized mode. It is worth noting that this value approaches the current state of the art in solid-state single-photon sources based on III/V QDs^42−44^ and widely outperforms monolayer-based triggered single-photon sources reported in any implementation.^15,39,45,46^ It furthermore is interesting to note that the congruence of directly measured first-lens brightness and theoretically calculated source extraction efficiency suggests that the internal quantum efficiency of our emitters approaches unity. We believe that this encouraging result was facilitated by the combination of utilizing high-quality TMDC materials and capping via hexagonal boron nitride, the resonant coupling to the photonic cavity, and finally the applied quasi-resonant pumping scheme, which does not allow for losses via high-momentum free-exciton states or relaxation into long-lived dark exciton states. A full set of measurements for a second WSe_2_ QD including cavity tuning, degree of linear polarization, first-lens brightness, and single photon purity can be found in Section S4 of the Supporting Information.

The final benchmark of the quantum-optical properties of our TMDC single-photon source is the temporal coherence of the emitted single-photon wavepackets, reflected in their capability to display genuine quantum interference. This property is of capital importance for quantum applications, since it guarantees the capability of photons to interfere and, thus, propagate entanglement along quantum nodes. It is furthermore of profound fundamental interest since such quantum interference from atomically thin emitters has not been observed thus far.

We implement the two-photon Hong–Ou–Mandel (HOM) interference in a path-unbalanced Mach–Zehnder interferometer (see the scheme of the setup in Figure S2 of the Supporting Information), interfering two photon wavepackets successively emitted by the source (with an initial temporal delay of 13 ns and eventually corrected by the delay in the interferometer). The quantum interference is extracted via the measurement of the second-order correlation function between the two detectors at the output of the interferometer (*g*_HOM_^(2)^). The perfect bosonic quantum interference features complete antibunching (*g*_HOM_^(2)^ = 0).

To quantify the quantum interference from the photons emitted by our source, we measure the HOM correlation between photons with parallel/orthogonal polarizations (*g*_HOM,HH_^(2)^/*g*_HOM,HV_^(2)^). Figure 3c shows the corresponding normalized correlation histograms, measured using the same excitation conditions as in the HBT measurement.

We compare the critical cases of photons of orthogonal polarization (HV) in the two interferometer arms versus those of parallel polarization (HH). As we reduce the width of the temporal selection window from 3 ns down to 1.1 ns—approaching the resolution limit of our detection setup—a significant difference between the parallel/orthogonal polarization correlations cases of the *g*_HOM_^(2)^ measurement arises consistently. Such an effect is depicted in the panel insets, where *g*_HOM,HH_^(2)^ and *g*_HOM,HV_^(2)^ correlations display different values beyond the standard deviation of the correlation peaks. This fact is further visualized in Figure 3d, where we depict the interference visibility *V* = (*g*_HOM,HV_^(2)^ – *g*_HOM,HH_^(2)^)/*g*_HOM,HV_^(2)^ as a function of the postselected temporal window.

The results on photon quantum interference manifest the presence of a substantial dephasing channel in the TMDC QD. From the modest interference visibility, we can estimate a dephasing time of 23.1 ps via a fit to the data in Figure 3d^47^ taking into account the instrument response function, which is consistent with previous studies of the line width of WSe_2_ QDs.^15^ We notice that without correcting for the finite *g*^(2)^ value in the HBT experiment, this yields a conservative estimate of *T*_2_. The dephasing most likely has its roots in rapid surface-induced charge noise and is only partly mitigated by the capping of our monolayer with hexagonal boron nitride. Thus, in order to further improve the coherence time of the QD emission, we suggest to further stabilize the charge environment via including graphene contacts to gate the system.^48^ We furthermore believe that it will be possible to boost the Purcell enhancement in our cavity beyond a factor of 10 via minimizing the mode volume (e.g., further closing the cavity) and slightly improving the cavity quality factor (e.g., by employing a top mirror with slightly enhanced reflectivity^42^ or resorting to nanoscale resonators).^49^

Harnessing the high first-lens brightness of 65% and purity of our source, it is readily applicable in quantum communication schemes which do not rely on quantum interference and entanglement, such as the BB84 protocol in urban networks.^39^ We furthermore believe that our implementation of the open cavity in a liquid-helium-free exchange-gas cryostat will inspire the single-photon source and quantum material cavity QED community toward accelerating the transition toward dry cryostats.

## Supporting Information Available

Additional details on the experimental setup and its calibration, sample preparation, numerical FDTD simulations, and additional data on the tunability of the open cavity (PDF)

## References

[ref1] Pan J.-W.; Chen Z.-B.; Lu C.-Y.; Weinfurter H.; Zeilinger A.; Żukowski M. Multiphoton Entanglement and Interferometry. Rev. Mod. Phys. 2012, 84 (2), 777–838. 10.1103/RevModPhys.84.777.

[ref2] Waks E.; Inoue K.; Santori C.; Fattal D.; Vuckovic J.; Solomon G. S.; Yamamoto Y. Quantum Cryptography with a Photon Turnstile. Nature 2002, 420 (6917), 762 10.1038/420762a. 12490939

[ref3] Senellart P.; Solomon G.; White A. High-Performance Semiconductor Quantum-Dot Single-Photon Sources. Nat. Nanotechnol. 2017, 12 (11), 1026–1039. 10.1038/nnano.2017.218. 29109549

[ref4] O’Brien J. L. Optical Quantum Computing. Science 2007, 318 (5856), 1567–1570. 10.1126/science.1142892. 18063781

[ref5] Wang H.; Qin J.; Chen S.; Chen M.-C.; You X.; Ding X.; Huo Y.-H.; Yu Y.; Schneider C.; Höfling S.; Scully M.; Lu C.-Y.; Pan J.-W. Observation of Intensity Squeezing in Resonance Fluorescence from a Solid-State Device. Phys. Rev. Lett. 2020, 125 (15), 153601 10.1103/PhysRevLett.125.153601. 33095635

[ref6] Aharonovich I.; Englund D.; Toth M. Solid-State Single-Photon Emitters. Nat. Photonics 2016, 10 (10), 631–641. 10.1038/nphoton.2016.186.

[ref7] Azzam S. I.; Parto K.; Moody G. Prospects and Challenges of Quantum Emitters in 2D Materials. Appl. Phys. Lett. 2021, 118 (24), 240502 10.1063/5.0054116.

[ref8] Turunen M.; Brotons-Gisbert M.; Dai Y.; Wang Y.; Scerri E.; Bonato C.; Jöns K. D.; Sun Z.; Gerardot B. D. Quantum Photonics with Layered 2D Materials. Nat. Rev. Phys. 2022, 4 (4), 219–236. 10.1038/s42254-021-00408-0.

[ref9] Koperski M.; Nogajewski K.; Arora A.; Cherkez V.; Mallet P.; Veuillen J.-Y.; Marcus J.; Kossacki P.; Potemski M. Single Photon Emitters in Exfoliated WSe2 Structures. Nat. Nanotechnol. 2015, 10 (6), 503–506. 10.1038/nnano.2015.67. 25938573

[ref10] He Y.-M.; Clark G.; Schaibley J. R.; He Y.; Chen M.-C.; Wei Y.-J.; Ding X.; Zhang Q.; Yao W.; Xu X.; Lu C.-Y.; Pan J.-W. Single Quantum Emitters in Monolayer Semiconductors. Nat. Nanotechnol. 2015, 10 (6), 497–502. 10.1038/nnano.2015.75. 25938571

[ref11] Chakraborty C.; Kinnischtzke L.; Goodfellow K. M.; Beams R.; Vamivakas A. N. Voltage-Controlled Quantum Light from an Atomically Thin Semiconductor. Nat. Nanotechnol. 2015, 10 (6), 507–511. 10.1038/nnano.2015.79. 25938569

[ref12] Srivastava A.; Sidler M.; Allain A. V.; Lembke D. S.; Kis A.; Imamoğlu A. Optically Active Quantum Dots in Monolayer WSe2. Nat. Nanotechnol. 2015, 10 (6), 491–496. 10.1038/nnano.2015.60. 25938570

[ref13] Tonndorf P.; Schmidt R.; Schneider R.; Kern J.; Buscema M.; Steele G. A.; Castellanos-Gomez A.; van der Zant H. S. J.; Michaelis de Vasconcellos S.; Bratschitsch R. Single-Photon Emission from Localized Excitons in an Atomically Thin Semiconductor. Optica 2015, 2 (4), 347 10.1364/OPTICA.2.000347.

[ref14] Kumar S.; Brotóns-Gisbert M.; Al-Khuzheyri R.; Branny A.; Ballesteros-Garcia G.; Sánchez-Royo J. F.; Gerardot B. D. Resonant Laser Spectroscopy of Localized Excitons in Monolayer WSe2. Optica 2016, 3 (8), 882 10.1364/OPTICA.3.000882.

[ref15] Luo Y.; Shepard G. D.; Ardelean J. V.; Rhodes D. A.; Kim B.; Barmak K.; Hone J. C.; Strauf S. Deterministic Coupling of Site-Controlled Quantum Emitters in Monolayer WSe2 to Plasmonic Nanocavities. Nat. Nanotechnol. 2018, 13 (12), 1137–1142. 10.1038/s41565-018-0275-z. 30374160

[ref16] Sortino L.; Zotev P. G.; Phillips C. L.; Brash A. J.; Cambiasso J.; Marensi E.; Fox A. M.; Maier S. A.; Sapienza R.; Tartakovskii A. I. Bright Single Photon Emitters with Enhanced Quantum Efficiency in a Two-Dimensional Semiconductor Coupled with Dielectric Nano-Antennas. Nat. Commun. 2021, 12 (1), 6063 10.1038/s41467-021-26262-3. 34663795 PMC8523570

[ref17] Wang Q.; Maisch J.; Tang F.; Zhao D.; Yang S.; Joos R.; Portalupi S. L.; Michler P.; Smet J. H. Highly Polarized Single Photons from Strain-Induced Quasi-1D Localized Excitons in WSe2. Nano Lett. 2021, 21 (17), 7175–7182. 10.1021/acs.nanolett.1c01927. 34424710 PMC8431731

[ref18] So J.-P.; Jeong K.-Y.; Lee J. M.; Kim K.-H.; Lee S.-J.; Huh W.; Kim H.-R.; Choi J.-H.; Kim J. M.; Kim Y. S.; Lee C.-H.; Nam S.; Park H.-G. Polarization Control of Deterministic Single-Photon Emitters in Monolayer WSe _2_. Nano Lett. 2021, 21 (3), 1546–1554. 10.1021/acs.nanolett.1c00078. 33502866

[ref19] Palacios-Berraquero C.; Kara D. M.; Montblanch A. R.-P.; Barbone M.; Latawiec P.; Yoon D.; Ott A. K.; Loncar M.; Ferrari A. C.; Atatüre M. Large-Scale Quantum-Emitter Arrays in Atomically Thin Semiconductors. Nat. Commun. 2017, 8 (1), 15093 10.1038/ncomms15093. 28530249 PMC5458119

[ref20] Branny A.; Kumar S.; Proux R.; Gerardot B. D. Deterministic Strain-Induced Arrays of Quantum Emitters in a Two-Dimensional Semiconductor. Nat. Commun. 2017, 8 (1), 15053 10.1038/ncomms15053. 28530219 PMC5458118

[ref21] Purcell E. M. Spontaneous Emission Probabilities at Radio Frequencies. In Confined Electrons and Photons; Burstein E., Weisbuch C., Eds.; Springer US: 1995; NATO ASI Series Vol. 340, p 839. 10.1007/978-1-4615-1963-8_40.

[ref22] Dousse A.; Lanco L.; Suffczyński J.; Semenova E.; Miard A.; Lemaître A.; Sagnes I.; Roblin C.; Bloch J.; Senellart P. Controlled Light-Matter Coupling for a Single Quantum Dot Embedded in a Pillar Microcavity Using Far-Field Optical Lithography. Phys. Rev. Lett. 2008, 101 (26), 267404 10.1103/PhysRevLett.101.267404. 19437672

[ref23] Sapienza L.; Davanço M.; Badolato A.; Srinivasan K. Nanoscale Optical Positioning of Single Quantum Dots for Bright and Pure Single-Photon Emission. Nat. Commun. 2015, 6 (1), 7833 10.1038/ncomms8833. 26211442 PMC4525159

[ref24] Schneider C.; Heindel T.; Huggenberger A.; Weinmann P.; Kistner C.; Kamp M.; Reitzenstein S.; Höfling S.; Forchel A. Single Photon Emission from a Site-Controlled Quantum Dot-Micropillar Cavity System. Appl. Phys. Lett. 2009, 94 (11), 111111 10.1063/1.3097016.

[ref25] Schwarz S.; Dufferwiel S.; Walker P. M.; Withers F.; Trichet A. A. P.; Sich M.; Li F.; Chekhovich E. A.; Borisenko D. N.; Kolesnikov N. N.; Novoselov K. S.; Skolnick M. S.; Smith J. M.; Krizhanovskii D. N.; Tartakovskii A. I. Two-Dimensional Metal–Chalcogenide Films in Tunable Optical Microcavities. Nano Lett. 2014, 14 (12), 7003–7008. 10.1021/nl503312x. 25375802 PMC4335560

[ref26] Najer D.; Söllner I.; Sekatski P.; Dolique V.; Löbl M. C.; Riedel D.; Schott R.; Starosielec S.; Valentin S. R.; Wieck A. D.; Sangouard N.; Ludwig A.; Warburton R. J. A Gated Quantum Dot Strongly Coupled to an Optical Microcavity. Nature 2019, 575 (7784), 622–627. 10.1038/s41586-019-1709-y. 31634901

[ref27] Greuter L.; Starosielec S.; Najer D.; Ludwig A.; Duempelmann L.; Rohner D.; Warburton R. J. A Small Mode Volume Tunable Microcavity: Development and Characterization. Appl. Phys. Lett. 2014, 105 (12), 121105 10.1063/1.4896415.

[ref28] Greuter L.; Starosielec S.; Kuhlmann A. V.; Warburton R. J. Towards High-Cooperativity Strong Coupling of a Quantum Dot in a Tunable Microcavity. Phys. Rev. B 2015, 92 (4), 045302 10.1103/PhysRevB.92.045302.

[ref29] Riedel D.; Söllner I.; Shields B. J.; Starosielec S.; Appel P.; Neu E.; Maletinsky P.; Warburton R. J. Deterministic Enhancement of Coherent Photon Generation from a Nitrogen-Vacancy Center in Ultrapure Diamond. Phys. Rev. X 2017, 7 (3), 031040 10.1103/PhysRevX.7.031040.

[ref30] Pallmann M.; Eichhorn T.; Benedikter J.; Casabone B.; Hümmer T.; Hunger D. A Highly Stable and Fully Tunable Open Microcavity Platform at Cryogenic Temperatures. APL Photonics 2023, 8 (4), 046107 10.1063/5.0139003.

[ref31] Ruelle T.; Jaeger D.; Fogliano F.; Braakman F.; Poggio M. A Tunable Fiber Fabry–Perot Cavity for Hybrid Optomechanics Stabilized at 4 K. Rev. Sci. Instrum. 2022, 93 (9), 095003 10.1063/5.0098140. 36182449

[ref32] Fontana Y.; Zifkin R.; Janitz E.; Rodríguez Rosenblueth C. D.; Childress L. A Mechanically Stable and Tunable Cryogenic Fabry–Pérot Microcavity. Rev. Sci. Instrum. 2021, 92 (5), 053906 10.1063/5.0049520. 34243336

[ref33] Casabone B.; Deshmukh C.; Liu S.; Serrano D.; Ferrier A.; Hümmer T.; Goldner P.; Hunger D.; de Riedmatten H. Dynamic Control of Purcell Enhanced Emission of Erbium Ions in Nanoparticles. Nat. Commun. 2021, 12 (1), 3570 10.1038/s41467-021-23632-9. 34117226 PMC8196009

[ref34] Vadia S.; Scherzer J.; Thierschmann H.; Schäfermeier C.; Dal Savio C.; Taniguchi T.; Watanabe K.; Hunger D.; Karraï K.; Högele A. Open-Cavity in Closed-Cycle Cryostat as a Quantum Optics Platform. PRX Quantum 2021, 2 (4), 040318 10.1103/PRXQuantum.2.040318.

[ref35] Merkel B.; Ulanowski A.; Reiserer A. Coherent and Purcell-Enhanced Emission from Erbium Dopants in a Cryogenic High- Q Resonator. Phys. Rev. X 2020, 10 (4), 041025 10.1103/PhysRevX.10.041025.

[ref36] Bogdanović S.; van Dam S. B.; Bonato C.; Coenen L. C.; Zwerver A.-M. J.; Hensen B.; Liddy M. S. Z.; Fink T.; Reiserer A.; Lončar M.; Hanson R. Design and Low-Temperature Characterization of a Tunable Microcavity for Diamond-Based Quantum Networks. Appl. Phys. Lett. 2017, 110 (17), 171103 10.1063/1.4982168.

[ref37] Iff O.; Tedeschi D.; Martín-Sánchez J.; Moczała-Dusanowska M.; Tongay S.; Yumigeta K.; Taboada-Gutiérrez J.; Savaresi M.; Rastelli A.; Alonso-González P.; Höfling S.; Trotta R.; Schneider C. Strain-Tunable Single Photon Sources in WSe2Monolayers. Nano Lett. 2019, 19, 6931 10.1021/acs.nanolett.9b02221. 31486648

[ref38] Simon C.; Afzelius M.; Appel J.; Boyer de la Giroday A.; Dewhurst S. J.; Gisin N.; Hu C. Y.; Jelezko F.; Kröll S.; Müller J. H.; Nunn J.; Polzik E. S.; Rarity J. G.; De Riedmatten H.; Rosenfeld W.; Shields A. J.; Sköld N.; Stevenson R. M.; Thew R.; Walmsley I. A.; Weber M. C.; Weinfurter H.; Wrachtrup J.; Young R. J. Quantum Memories: A Review Based on the European Integrated Project “Qubit Applications (QAP). Eur. Phys. J. D 2010, 58 (1), 1–22. 10.1140/epjd/e2010-00103-y.

[ref39] Gao T.; von Helversen M.; Antón-Solanas C.; Schneider C.; Heindel T. Atomically-Thin Single-Photon Sources for Quantum Communication. Npj 2D Mater. Appl. 2023, 7 (1), 1–9. 10.1038/s41699-023-00366-4.

[ref40] Tripathi L. N.; Iff O.; Betzold S.; Dusanowski Ł.; Emmerling M.; Moon K.; Lee Y. J.; Kwon S.-H.; Höfling S.; Schneider C. Spontaneous Emission Enhancement in Strain-Induced WSe2Monolayer-Based Quantum Light Sources on Metallic Surfaces. ACS Photonics 2018, 5 (5), 1919–1926. 10.1021/acsphotonics.7b01053.

[ref41] Kern J.; Trügler A.; Niehues I.; Ewering J.; Schmidt R.; Schneider R.; Najmaei S.; George A.; Zhang J.; Lou J.; Hohenester U.; Michaelis de Vasconcellos S.; Bratschitsch R. Nanoantenna-Enhanced Light–Matter Interaction in Atomically Thin WS2. ACS Photonics 2015, 2 (9), 1260–1265. 10.1021/acsphotonics.5b00123.

[ref42] Tomm N.; Javadi A.; Antoniadis N. O.; Najer D.; Löbl M. C.; Korsch A. R.; Schott R.; Valentin S. R.; Wieck A. D.; Ludwig A.; Warburton R. J. A Bright and Fast Source of Coherent Single Photons. Nat. Nanotechnol. 2021, 16 (4), 399–403. 10.1038/s41565-020-00831-x. 33510454

[ref43] Unsleber S.; He Y.-M.; Gerhardt S.; Maier S.; Lu C.-Y.; Pan J.-W.; Gregersen N.; Kamp M.; Schneider C.; Höfling S. Highly Indistinguishable On-Demand Resonance Fluorescence Photons from a Deterministic Quantum Dot Micropillar Device with 74% Extraction Efficiency. Opt. Express 2016, 24 (8), 8539 10.1364/OE.24.008539. 27137291

[ref44] Somaschi N.; Giesz V.; De Santis L.; Loredo J. C.; Almeida M. P.; Hornecker G.; Portalupi S. L.; Grange T.; Antón C.; Demory J.; Gómez C.; Sagnes I.; Lanzillotti-Kimura N. D.; Lemaítre A.; Auffeves A.; White A. G.; Lanco L.; Senellart P. Near-Optimal Single-Photon Sources in the Solid State. Nat. Photonics 2016, 10 (5), 340–345. 10.1038/nphoton.2016.23.

[ref45] Flatten L. C.; Weng L.; Branny A.; Johnson S.; Dolan P. R.; Trichet A. A. P.; Gerardot B. D.; Smith J. M. Microcavity Enhanced Single Photon Emission from Two-Dimensional WSe2. Appl. Phys. Lett. 2018, 112 (19), 191105 10.1063/1.5026779.

[ref46] He Y.-M.; Iff O.; Lundt N.; Baumann V.; Davanco M.; Srinivasan K.; Höfling S.; Schneider C. Cascaded Emission of Single Photons from the Biexciton in Monolayered WSe2. Nat. Commun. 2016, 7 (1), 13409 10.1038/ncomms13409. 27830703 PMC5109589

[ref47] Bylander J.; Robert-Philip I.; Abram I. Interference and Correlation of Two Independent Photons. Eur. Phys. J. - At. Mol. Opt. Plasma Phys. 2003, 22 (2), 295–301. 10.1140/epjd/e2002-00236-6.

[ref48] Brotons-Gisbert M.; Branny A.; Kumar S.; Picard R.; Proux R.; Gray M.; Burch K. S.; Watanabe K.; Taniguchi T.; Gerardot B. D. Coulomb Blockade in an Atomically Thin Quantum Dot Coupled to a Tunable Fermi Reservoir. Nat. Nanotechnol. 2019, 14 (5), 442–446. 10.1038/s41565-019-0402-5. 30858522

[ref49] Iff O.; Buchinger Q.; Moczała-Dusanowska M.; Kamp M.; Betzold S.; Davanco M.; Srinivasan K.; Tongay S.; Antón-Solanas C.; Höfling S.; Schneider C. Purcell-Enhanced Single Photon Source Based on a Deterministically Placed WSe2Monolayer Quantum Dot in a Circular Bragg Grating Cavity. Nano Lett. 2021, 21 (11), 4715–4720. 10.1021/acs.nanolett.1c00978. 34048254 PMC10573669

