# Supplementary material for: Monolayer-Based Single-Photon Source in a Liquid-Helium-Free Open Cavity Featuring 65% Brightness and Quantum Coherence
Source: Nano Lett. 2023 Sep 9;23(18):8683–9. doi: 10.1021/acs.nanolett.3c02584 (PMC10540255; doi:10.1021/acs.nanolett.3c02584)
Supplement: Supplementary file 1 — nl3c02584_si_001.pdf [file nl3c02584_si_001.pdf]

Supporting Information:

Monolayer-based single photon source in a liquid-helium-free open cavity featuring 65% brightness and quantum coherence

Jens-Christian Drawer[1,‡], Victor Nikolaevich Mitryakhin[1, ‡], Hangyong Shan[1, ‡], Sven Stephan[1,2], Moritz Gittinger[1], Lukas Lackner[1], Bo Han[1], Gilbert Leibeling[3], Falk Eilenberger[3], Rounak Banerjee[4], Sefaattin Tongay[4], Kenji Watanabe[5], Takashi Taniguchi[6], Christoph Lienau[1], Martin Silies[2], Carlos Anton-Solanas[7], Martin Esmann[1], and Christian Schneider[1,]*

[1]*Institute of Physics, Carl von Ossietzky University Oldenburg, 26129 Oldenburg, Germany*

[2]*University of Applied Sciences Emden/Leer, 26723 Emden, Germany*

[3]*Institute of Applied Physics, Abbe Center of Photonics, Friedrich Schiller University Jena, 07743 Jena, Germany; Fraunhofer-Institute for Applied Optics and Precision Engineering IOF, 07743 Jena, Germany; Max-Planck-School of Photonics, 07743 Jena, Germany*

[4]*Materials Science and Engineering, School for Engineering of Matter, Transport, and Energy, Arizona State University, Tempe, 85287, Arizona, USA*

[5]*Research Center for Functional Materials, National Institute for Materials Science, 1-1 Namiki, Tsukuba 305-0044, Japan*

[6]*International Center for Materials Nanoarchitectonics, National Institute for Materials Science, 1-1 Namiki, Tsukuba 305-0044, Japan*

[7]*Depto. de Física de Materiales, Instituto Nicolás Cabrera, Instituto de Física de la Materia Condensada, Universidad Autónoma de Madrid, 28049 Madrid, Spain*

Supplementary section S1: Sample preparation and experimental setup details

Sample preparation. The sample is a Fabry-Perot type cavity consisting of two separated mirror pieces. The bottom part of the cavity is a distributed Bragg reflector made of 10 pairs of TiO_2/SiO_2 layers. The thicknesses of the layers are 85 nm and 131 nm respectively. This configuration corresponds to a high reflectivity region (so called stop band) centered at 755 nm. On top of this DBR, an atomically thin layer of WSe_2 is placed via the dry-gel stamping method. A layer of hexagonal boron nitride serving as capping layer is transferred onto the bottom part of the sample. The upper part of the open cavity consists of a SiO_2 substrate, into which a square mesa with dimensions of 100 µm x 100 µm is milled. A focused ion beam (FIB, FEI Helios 600i) was used to mill lenses with diameters of 3, 3.5, 4, 5 and 6 µm and identical depth of 300 nm into the mesa surface. Finally, a 33 nm layer of gold was evaporated onto this structure. Since the upper gold mirror prevents visualization of the bottom mirror, a 20 µm x 30 µm window was etched by FIB, removing the gold on the mesa. The top and bottom mirrors are mounted onto separate motorized stages featuring nanopositioning in XYZ directions. The whole device is located in a low-vibration closed cycle cryostat, allowing high stability of the cavity alignment at low temperatures.

Optical Spectroscopy settings. All measurements have been conducted in a confocal microscope setup with excitation and collection through a lens in the top part of the sample. A simplified partial sketch of the setup is discussed in Fig. S1 below. The collected emission is filtered by a longpass filter ensuring that the laser reflected into the path in not present in the spectrum. The emission is then guided with a set of mirrors and projected onto the slit of a monochromator, diffracted and recorded by a Peltier-cooled charge coupled device.

To isolate the single photon emission, the spectrum is accordingly filtered with a set of tunable (within the range of 710-800 nm) shortpass and longpass filters achieving bandpass windows down to 2-3 meV. In the HBT setup configuration, the emission is coupled via a zoom collimator to a single mode fiber that is then connected to a 50:50 fiber beam splitter. Its two outputs are each connected to an avalanche photodiode as a single photon detector. A time-correlation device receives the signals from both APDs and the coincidences of the events within a certain bin size are recorded. The setup configuration for the study of Hong-Ou-Mandel type interferences is detailed in Fig. S2.

Open cavity configuration in a closed-cycle cryostat. In Fig. S1, we depict the schematic implementation of the open cavity system. The implementation shares conceptual similarities with previous geometries utilized in helium bath cryostats [1].

We designed and customized the open cavity system to operate it in a liquid helium-free closed-cycle cryostat (AttoDry 1000) at a temperature of 3.2 K. The cryostat is equipped with a superconducting magnet reaching fields up to 9 T (not utilized in this work). The adapted design is optimized to passively reduce the vertical vibrations between the cavity mirrors imposed by the compressor pulse-tube between the cavity mirrors. So far, other approaches in closed-cycle cryostats used actively-locked open cavities (such as Refs. [2–8]), considerably increasing the complexity and experimental resources of the setup.

We use a solid, cylindrical titanium housing enclosing the internal XYZ positioners (Attocube). The bottom DBR is mounted on top of it. The distance between the top and bottom mirrors (cavity length) is precisely controlled by the internal Z positioner (Attocube). Single photon emitters on the bottom mirror are aligned to micro-lens structures of the top mirror via the internal XY positioners (Attocube).

A titanium cover holds the top gold mirror (pasted to the internal surface), and the cover presents a small diameter hole (the micro-lens structures of the top mirror are aligned with the centre of this cover aperture), through which the excitation and collection of photons is implemented. We use external XY positioners to horizontally align the assembly (i.e., internal XYZ positioners, bottom mirror, top mirror, and titanium cover), relative to the excitation laser spot. The first lens can be adjusted vertically by an external Z positioner. The whole motor set is described in the following section.

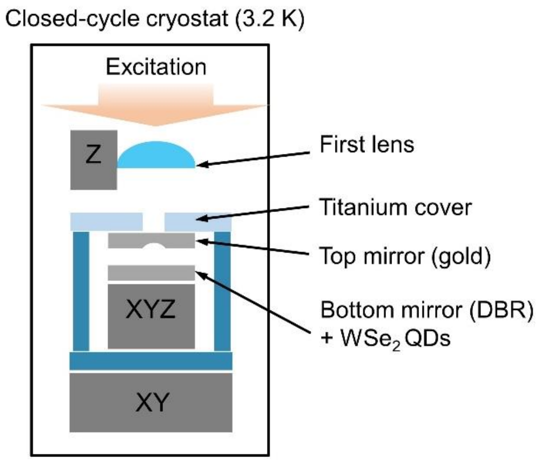

Figure S1. Sketch of the open cavity system in a liquid helium-free closed-cycle cryostat.

Photoluminescence measurements. Excitation is done either by a Coherent Mira Optima 900-F mode-locked Ti:Sapphire laser or a 532 nm diode laser. The bottom mirror with the deposited monolayer is mounted on the internal XYZ positioners. The cavity assembly is mounted on external XY positioners in a closed-cycle cryostat, operating at 3.2 K. The first lens above the sample is a Thorlabs 354105-B with NA = 0.6 mounted on a Z positioner inside the cryostat. The sample photoluminescence is recorded by a charge coupled device (CCD), model Andor iKon-M 934 Series, attached to a spectrometer, model Andor Shamrock SR-500i. The CCD exposure time is set to 1 s.

Correlation and time-resolved measurements. The single photon emission line is spectrally filtered by a set of short- and long-pass filters. The resulting signal is then coupled into a single mode fiber (780HP). For the Hanbury-Brown-Twiss (HBT) measurement, its output is coupled to a 50:50 single mode fiber beam splitter. For the Hong-Ou-Mandel (HOM) measurement, both free space and fiber beam splitters are used. The sketch of the setup for HOM measurements is shown in Fig. S2. The fiber beam splitter outputs are connected to two avalanche photodiode detectors (APDs) and their electronic outputs are connected to a time tagger (model quTAG) where the two-photon correlation histograms are reconstructed. The timing jitter of the APDs is specified as 500 ps.

In the HBT measurements, the measured second order correlation function is a convolution of the pristine $g^{(2)}$ function and the system response function F. Therefore, we perform a

deconvolution to obtain the pristine $g^{(2)}$ function. For this purpose, we couple the pulsed laser (2 ps pulse width) into the HBT setup and determine the system response function F from the collected correlation histogram.

To study the photon purity, the HBT correlation histogram recorded by the time tagger is fitted with a series of double exponential decay functions, equidistantly spaced by T (laser repetition period T =13.12 ns), with equal decay time a and individual peak heights d_i, convolved with the measured system response function F, which is normalized to $\int_{-\infty}^{\infty} \mathrm{F}\,(\tau)\mathrm{d}\tau = 1$. A constant C is added to account for the background of the correlation histogram. This gives the model function

$$\Pi(\tau) = C + \sum_i d_i \cdot \left(\mathrm{F} * e^{-|\cdot|/a}\right)(\tau + i \cdot T), \tag{1}$$

which is fitted to the entire measured correlation histogram. The relevant parameters obtained from the fit to the measured histogram are $a = 2773 \pm 19$ ps, $C = 118 \pm 5$, and $\bar{d}_{i \neq 0} = 1255.7 \pm 1.1$.

We normalize the measured correlation histogram with the average height of the uncorrelated peaks and get the $g^{(2)}$ function as displayed in Fig. 3a of the main text. To obtain the value of $g^{(2)}(0)$, we divide the height of the zero-delay peak d_0 by the average height of the uncorrelated peaks $\bar{d}_{i \neq 0}$.

For the analysis of the second order correlation function in the HOM measurement, the counts, integrated over the respective temporal post-selection window, are taken as peak values d_i and the normalization is performed with respect to $\bar{d}_{i \neq 0, \pm 1}$, i.e., apart from the zero-delay peak, we additionally discard the peaks at ± 13.12 ns delay from the uncorrelated-peak average.

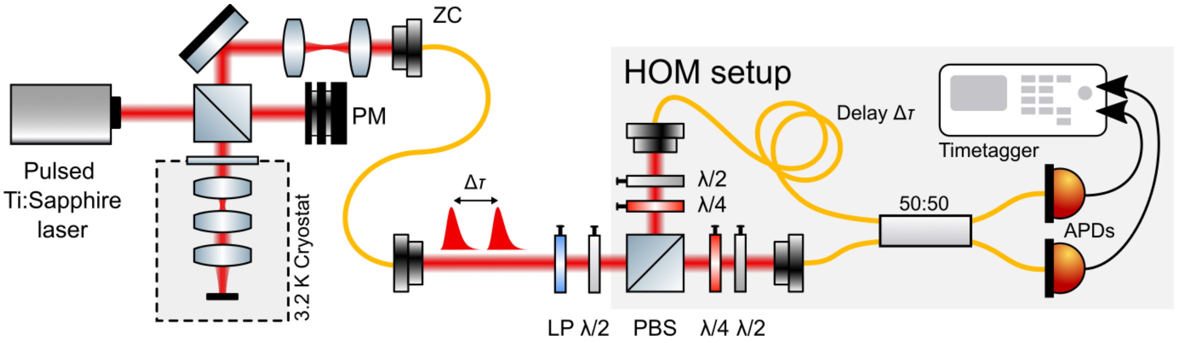

Figure S2. Sketch of the HOM interferometer setup. The sample is placed in a closed-cycle cryostat and kept at a temperature of 3.2 K while irradiated by a pulsed Ti:Sapphire laser (Coherent Mira Optima 900-F). The emission from the sample is then spectrally filtered and guided through a zoom collimator (ZC) to the Mach-Zehnder interferometer. In the collection path, a pair of lenses is set up as a telescope to adjust the beam size and improve the efficiency of the fiber coupling. At the input of the interferometer, a linear polarizer (LP) and a half-waveplate are placed in front of a polarizing beam splitter (PBS). This allows the two arms of the interferometer to be balanced in power. The output beams from the PBS then pass through fine polarization control optics (a quarter- and a half-waveplate) that compensate for differences in polarization at the fiber beamsplitter induced by propagation in the fibers. One of the half waveplates is used to rotate the polarization of one arm with respect to the other to achieve the cases of parallel (HH) or orthogonal (HV) polarization. In addition, one arm is delayed by the repetition period of the excitation laser. PM: power meter; ZC: zoom collimator; LP: linear polarizer; PBS: polarizing beam splitter; λ/2 (λ/4): half-waveplate (quarter-waveplate); APDs: avalanche photodiodes.

Supplementary section S2: FDTD simulation

The electromagnetic field inside the cavity has been simulated via placing a dipole emitter on the bottom mirror of the cavity, utilizing the commercial software Lumerical. Monitoring the power radiated from the dipole throughout the structure allowed us to derive the Purcell factor, the transmission coefficient of the upper part of the cavity and to acquire the field distribution for specific wavelengths.

The bottom mirror of the cavity is simulated as 10 alternating SiO_2/TiO_2 layers placed on a substrate with refractive index n = 1.5 (glass), starting with TiO_2. The refractive indices and layer thicknesses are taken from data provided by the mirror manufacturer Laseroptik GmbH: $n_{SiO2} = 1.45$, $d_{SiO2} = 131$ nm, $n_{TiO2} = 2.28$, $d_{TiO2} = 85$ nm. The WSe_2 flake is simulated as a thin layer of a high refractive index dielectric with $nWSe_2 = 4.3$, i.e., the refractive index at the energy of the emitter. A 5 nm thick layer of hBN with $n_{hBN} = 2.25$ is placed on top. The top mirror of the cavity consists of a spherical lens with a maximum depth of 300 nm and a diameter of 5 μm, etched into a substrate with n = 1.5 (glass). The lens is, as in the experiment, covered with a thin gold layer of 33 nm thickness. The dielectric function of gold at cryogenic temperatures is taken from Ref. [9]

The cavity modes are excited by a broadband dipole emitter that is placed inside the WSe_2 layer, with the dipole moment oriented along the x-axis. The dipole is surrounded by a set of monitors to measure the total power radiated by the dipole to record the energy-dependent Purcell factor. The spatial field profiles are recorded in the xz-plane. Radiation leaving the cavity, either through the top or bottom, is recorded by transmission monitors to compute the extraction efficiency. The transmission monitors are located 400 nm above the gold surface and 200 nm below the DBR.

The total simulation size is 7.5x7.5x8.8 μm^3 and the simulation runs for 3.5 ps such that the fields inside the cavity have sufficiently decayed. A spatially variable meshing is applied with cell sizes ranging from approx. 50 nm in free-space to 2 nm for the WSe_2 layer and 3 nm for the gold film of the top mirror. Symmetric and anti-symmetric boundary conditions are applied in order to reduce the run-time and memory demand of the simulation.

The simulations allow extracting the energy-dependent Purcell factor (i.e., the acceleration of the spontaneous emission of the emitter) and the cavity transmission (relevant for the double resonance excitation conditions that we put in place) through the 5 μm diameter lens etched in the top mirror. In the investigated spectral range, we observe the formation of Fabry-Perot modes, which are identified by peaks in both transmission and Purcell factor. For the mode labelled III in Fig. 1c of the main text, we study the spatial mode profile by analyzing the real space distribution of the electric field intensity (Fig. 1c, bottom). It shows a strong confinement of the electromagnetic field in the xy-plane to a diameter of ~2 μm due to the micro-lens structure. For this particular mode we find that 77 % of the emitted light is directed to the top, of which 65 % is transmitted through the top mirror and 12 % is absorbed by the gold. Transmission through the bottom mirror amounts to 6 %, with the remaining 17 % accounting for scattering losses to the sides.

Supplementary section S3: Additional data

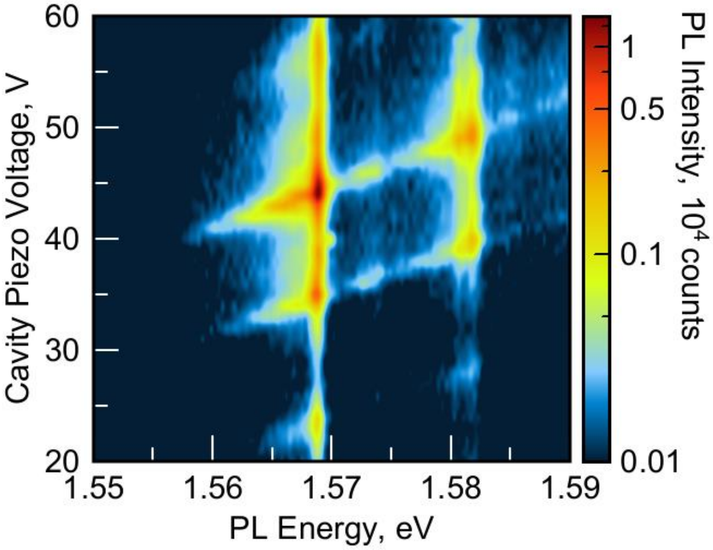

Figure S3. Colormap of PL spectra under 532 nm laser excitation. The data is reproduced from Fig. 2b in the main text, which is plotted with a linear false color-scale. To clearly exhibit the cavity modes, here we encode the data with a logarithmic color-scale. The bright inclined lines represent the optical modes of the open cavity device.

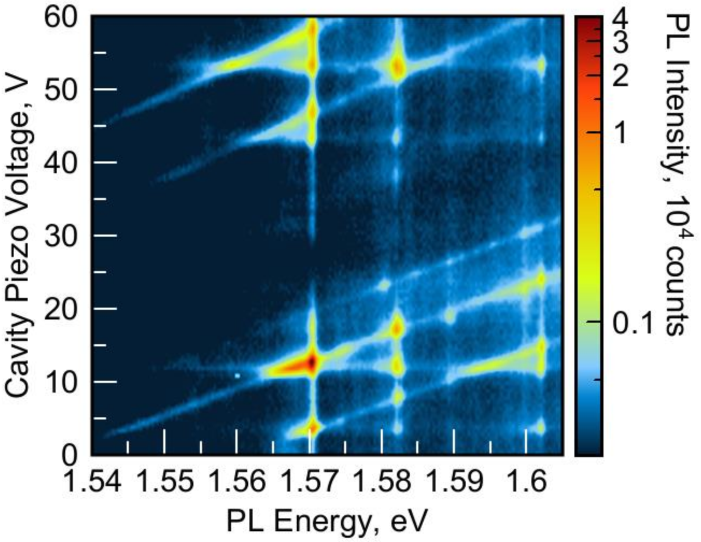

Figure S4. Colormap of PL spectra as a function of the cavity detuning and the emission energy, excited by 740 nm pulsed laser. In comparison to the case of 532 nm excitation (Fig. S3), the newly emerging horizontal features are observed (e.g., for cavity length corresponding to 54 and 12 V DC applied to the piezo of one of the Z direction nanopositioners). This indicates that a cavity mode is in resonance with the fixed wavelength laser excitation. When the excitation laser and the PL emission simultaneously match in frequency with a cavity mode, the system is in double resonance, for which the PL enhancement is most pronounced.

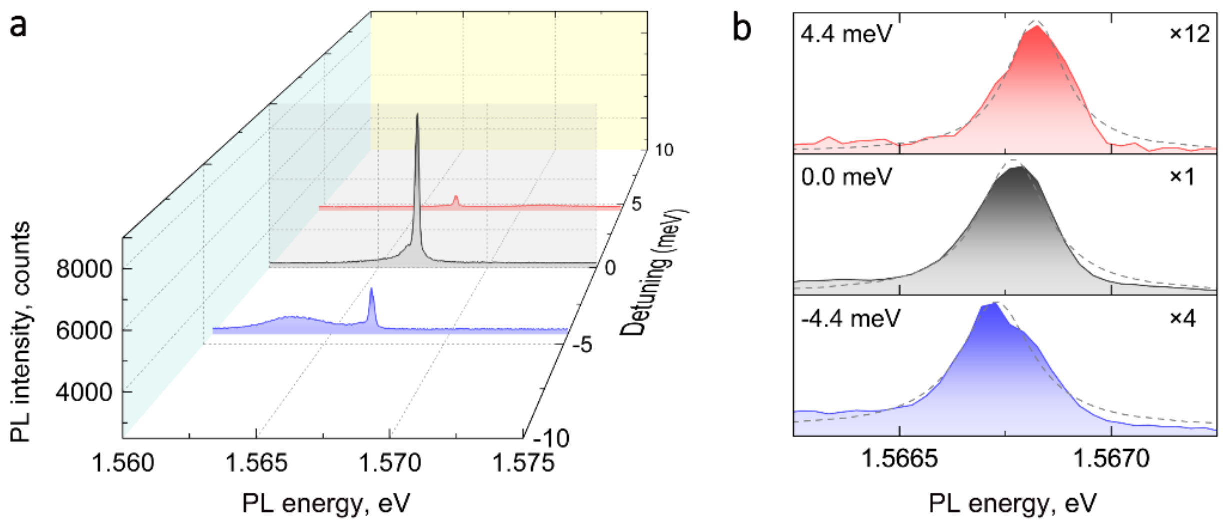

Figure S5. Representative spectra at different detuning values under double resonance condition. **a** PL spectra of single photon emission, at resonance (gray), positive (red), and negative (blue) detuning. Laser excitation at 740 nm is resonant with one of the optical modes of the open cavity. **b** Zoomed-in spectra of the single photon emission peak area in the spectra shown in panel a. The peaks are fitted with a Lorentzian function (dashed lines). The resulting linewidth is 200 μeV (extracted for zero detuning).

Experimental setup calibration. In Tab. S1 we list all contributions to the optical losses in our experimental setup.

	Value	Abs. Error
Cryostat (cryostat window, 3 lenses) and beamsplitter transmission (*)	22.87 %	0.05 %
Cryostat (cryostat window, 3 lenses) transmission	45.22 %	0.10 %
Beamsplitter transmission	50.57 %	0.14 %
Free space path transmission (2 lenses, 7 mirrors)	29.29 %	0.14 %
Spectral filter transmission and fiber coupling efficiency (*)	50.4 %	1.9 %
Spectral filter transmission	79.9 %	2.0 %
Fiber coupling efficiency	63.0 %	2.2 %
Avalanche photodiode efficiency	64.3 %	2.2 %
**Total efficiency**	**2.17 %**	**0.11 %**
Dead time corrected count rate of the APD	1080 kHz	40 kHz
Excitation laser repetition rate	76227.93 kHz	0.18 kHz
**First-lens brightness**	**65 %**	**4 %**

Table S1. Calibration for losses in the optical setup. (*): Combined measurement to reduce the error.

Supplementary section S4: Investigation of a second quantum emitter (QD2)

In the following we present a full measurement dataset of a second WSe_2 quantum dot (labelled QD2, whereas the QD shown in the main text is referred to as QD1 within this section). Figure S6 and Table S2 summarize our findings: The influence of a continuously varied cavity length on the sample PL is shown as a color heat map (Fig. S6 a), with two transverse cavity modes of same longitudinal order highlighted by a guide to the eye. Like QD1, QD2 emits single photons with near-perfect DOLP of 98 ± 5 % (Fig. S6 b). We attribute this observation to the QDs being strain-defined through wrinkles in the WSe_2 flake. QD2 also shows high purity of the single photon emission characterized by a second order autocorrelation of $g^{(2)}(0) = 9.9 \pm 0.7$ % (Fig. S6 c). Since QD2 emits at a wavelength around 774 nm, we repeated the calibration of the setup transmission losses. The individual contributions to the overall loss budget are tabulated in Table S2. This calibration results in a calibrated first-lens brightness for QD2 of 51 ± 5 % slightly below QD1.

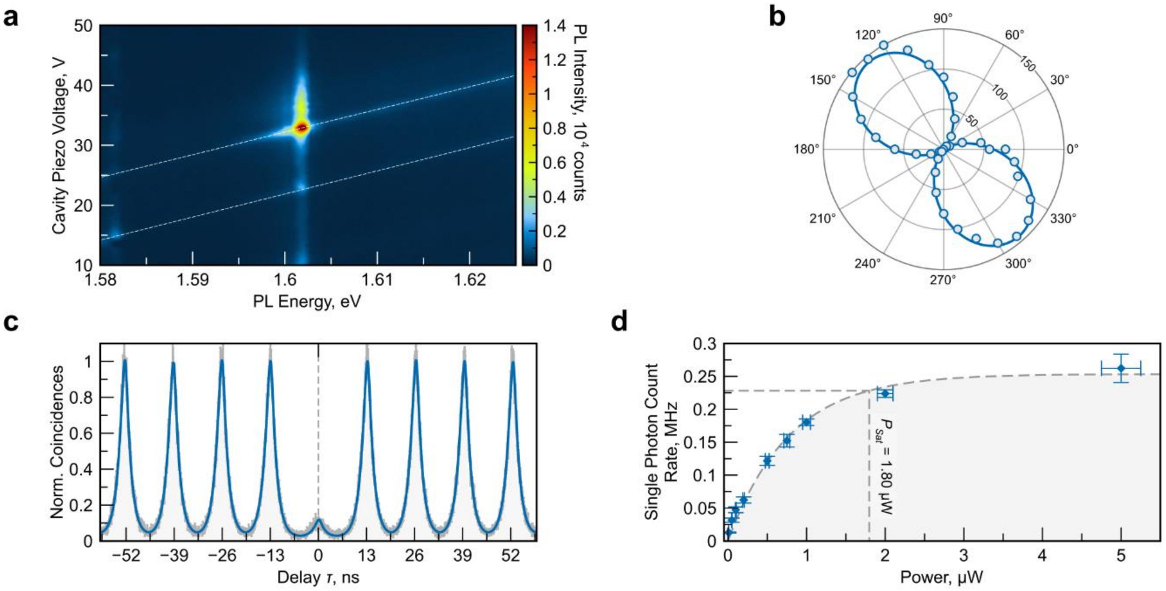

Figure S6. Characteristic features of QD2. a Colormap of PL spectra when tuning the cavity optical length while the sample is strongly excited above bandgap and outside of the stopband of the microcavity by a 532 nm continuous wave laser. Cavity modes are highlighted by dashed lines. **b** Polar plot of polarization-resolved PL intensity of the emission under 532 nm continuous wave excitation. The sinusoidal fit reveals a degree of linear polarization of 98 ± 5 %. **c** Second order autocorrelation function of single photons measured in an HBT experiment with 76.2 MHz pulsed excitation in the saturation regime. The data is fitted by a double exponential decay convoluted with the system response function. The relevant parameters (as introduced in Section S1) obtained from the fit to the measured histogram are $a = 1439 \pm 7$ ps, $C = 7.0 \pm 0.4$, and $\bar{d}_{i\neq 0} = 424.2 \pm 0.5$. **d** Brightness of the source as a function of optical pump power measured before focusing onto the sample. Errors are the standard errors as the result of averaging over several samples for each point. The measured single photon count rate at 5 μW pump power corresponds to a calibrated first lens brightness of 51 $\pm$ 5 %.

	Value	Abs. Error
Cryostat (cryostat window, 3 lenses) and beamsplitter transmission (*)	19.43 %	0.07 %
Cryostat (cryostat window, 3 lenses) transmission	37.22 %	0.14 %
Beamsplitter transmission	52.19 %	0.16 %
Free space path transmission (2 lenses, 7 mirrors)	29.7 %	0.6 %
Spectral filter transmission and fiber coupling efficiency (*)	17.4 %	0.8 %
Spectral filter transmission	70.2 %	2.5 %
Fiber coupling efficiency	24.9 %	1.1 %
Avalanche photodiode efficiency (**)	67.3 %	2.3 %
**Total efficiency**	**0.68 %**	**0.04 %**
Dead time corrected count rate of the APD	262 kHz	22 kHz
Excitation laser repetition rate	76227.93 kHz	0.18 kHz
**First-lens brightness**	**51 %**	**5 %**

Table S2. Calibration for losses in the optical setup for QD2. (*): Combined measurement to reduce the error. (**): Derived from the measured 790 nm sensitivity in Tab. S1 by scaling according to the manufacturer-specified spectral sensitivity.

References

(1) Tomm, N.; Javadi, A.; Antoniadis, N. O.; Najer, D.; Löbl, M. C.; Korsch, A. R.; Schott, R.; Valentin, S. R.; Wieck, A. D.; Ludwig, A.; Warburton, R. J. A Bright and Fast Source of Coherent Single Photons. *Nat. Nanotechnol.* **2021**, *16* (4), 399–403. https://doi.org/10.1038/s41565-020-00831-x.

(2) Pallmann, M.; Eichhorn, T.; Benedikter, J.; Casabone, B.; Hümmer, T.; Hunger, D. A Highly Stable and Fully Tunable Open Microcavity Platform at Cryogenic Temperatures. *APL Photonics* **2023**, *8* (4), 046107. https://doi.org/10.1063/5.0139003.

(3) Ruelle, T.; Jaeger, D.; Fogliano, F.; Braakman, F.; Poggio, M. A Tunable Fiber Fabry–Perot Cavity for Hybrid Optomechanics Stabilized at 4 K. *Rev. Sci. Instrum.* **2022**, *93* (9), 095003. https://doi.org/10.1063/5.0098140.

(4) Fontana, Y.; Zifkin, R.; Janitz, E.; Rodríguez Rosenblueth, C. D.; Childress, L. A Mechanically Stable and Tunable Cryogenic Fabry–Pérot Microcavity. *Rev. Sci. Instrum.* **2021**, *92* (5), 053906. https://doi.org/10.1063/5.0049520.

(5) Casabone, B.; Deshmukh, C.; Liu, S.; Serrano, D.; Ferrier, A.; Hümmer, T.; Goldner, P.; Hunger, D.; de Riedmatten, H. Dynamic Control of Purcell Enhanced Emission of Erbium Ions in Nanoparticles. *Nat. Commun.* **2021**, *12* (1), 3570. https://doi.org/10.1038/s41467-021-23632-9.

(6) Vadia, S.; Scherzer, J.; Thierschmann, H.; Schäfermeier, C.; Dal Savio, C.; Taniguchi, T.; Watanabe, K.; Hunger, D.; Karraï, K.; Högele, A. Open-Cavity in Closed-Cycle Cryostat as a Quantum Optics Platform. *PRX Quantum* **2021**, *2* (4), 040318. https://doi.org/10.1103/PRXQuantum.2.040318.

(7) Merkel, B.; Ulanowski, A.; Reiserer, A. Coherent and Purcell-Enhanced Emission from Erbium Dopants in a Cryogenic High- Q Resonator. *Phys. Rev. X* **2020**, *10* (4), 041025. https://doi.org/10.1103/PhysRevX.10.041025.

(8) Bogdanović, S.; van Dam, S. B.; Bonato, C.; Coenen, L. C.; Zwerver, A.-M. J.; Hensen, B.; Liddy, M. S. Z.; Fink, T.; Reiserer, A.; Lončar, M.; Hanson, R. Design and Low-Temperature Characterization of a Tunable Microcavity for Diamond-Based Quantum Networks. *Appl. Phys. Lett.* **2017**, *110* (17), 171103. https://doi.org/10.1063/1.4982168.

(9) Weaver, J. H.; Krafka, C.; Lynch, D. W.; Koch, E. E. Optical Properties of Metals. *Appl. Opt.* **1981**, *20* (7), 1124_1-1125. https://doi.org/10.1364/AO.20.1124_1.
